# The Physician’s Dose and Symptom Book

**Published:** 1869-03

**Authors:** 


### THE PHYSICIAN'S DOSE AND SYMPTOM BOOK,

Containing the Doses and uses of all the principal articles of the *Materia Medica*, and officinal preparations, etc., etc.

By JOSEPH H. WYTHES, A. M., M. D.

EIGHTH EDITION.

"This little work was compiled for the assistance of students, and to furnish a *vade mecum* for the general practitioner, which would save the trouble of referring to larger and more elaborate works." This is eminently a *multum in parvo*, and when once employed by any one in any branch of medical practice, will be regarded as indispensable. For sale by R. W. Carroll & Co.

